# Effect of an Intermediate-Frequency Magnetic Field of 23 kHz at 2 mT on Chemotaxis and Phagocytosis in Neutrophil-Like Differentiated Human HL-60 Cells

**DOI:** 10.3390/ijerph110909649

**Published:** 2014-09-17

**Authors:** Shin Koyama, Eijiro Narita, Naoki Shinohara, Junji Miyakoshi

**Affiliations:** Laboratory of Applied Radio Engineering for Humanosphere, Research Institute for Sustainable Humanosphere, Kyoto University, Gokasho, Uji, Kyoto 611-0011, Japan; E-Mails: shin_koyama@rish.kyoto-u.ac.jp (S.K.); narita.eijirou.4e@kyoto-u.ac.jp (E.N.); shino@rish.kyoto-u.ac.jp (N.S.)

**Keywords:** induction-heating (IH) cooktop, intermediate-frequency (IF) magnetic field, neutrophil, chemotaxis, phagocytosis

## Abstract

Public concerns about potential health risks of intermediate-frequency (IF) electromagnetic fields are increasing, especially as the use of induction-heating cooktops has spread extensively in Japan and Europe. In order to investigate the properties of IF electromagnetic fields, we examined the effect of exposure to a 23-kHz IF magnetic field of 2 mT for 2, 3, or 4 h on neutrophil chemotaxis and phagocytosis using differentiated human HL-60 cells. Compared with sham exposure, exposure to the IF magnetic field had no effect on neutrophil chemotaxis or phagocytosis. Previous studies demonstrated that exposure to a 23-kHz IF magnetic field of 2 mT (about 74-times the maximum value recommended by the International Commission for Nonionizing Radiation Protection guidelines) may affect the first-line immune responses in humans. To our knowledge, this is the first study to evaluate the effects of IF magnetic fields on cellular immune responses. We found that exposure to an IF magnetic field of 2 mT has minimal if any effect on either the chemotaxis or phagocytic activity of neutrophil-like human HL-60 cells.

## 1. Introduction

Intermediate-frequency (IF) electromagnetic fields (300 Hz to 10 MHz) generated by induction-heating (IH) cooktops are now widespread in domestic kitchens because of the replacement of gas for cooking in Japan and Europe. IH cooktops generate magnetic fields at around 20 to 100 kHz, frequencies that are in the IF range. Unfortunately, research examining the potential health effects associated with exposure to IF magnetic fields is lacking. As concern regarding the potential health effects has risen, it has become necessary to investigate the risks of IF magnetic fields in more detail.

Several epidemiologic [1,2,3,4] and *in vivo* studies [5,6,7] of IF magnetic fields have been published. These studies have focused on the effects working with video-display terminals may have on women. In addition, some *in vitro* studies [8,9,10,11] of the effect of exposure to IF magnetic fields have been conducted in Japan since gas and electric stoves have been increasingly replaced by IH cooktops. Most IH cooktops use a 23-kHz magnetic field as the maximum output power frequency. We previously reported that 23-kHz IF magnetic fields have no detectable effects on human gene-expression profiles [8]. In that study, we used a 100 µT_rms_ IF magnetic field, which was approximately 16-times higher than the reference level for general public exposure to magnetic fields of 3 kHz to 10 MHz as stated in the 1998 International Commission on Nonionizing Radiation Protection (ICNIRP) guidelines [12]. However, recently (2013) updated ICNIRP guidelines revised the reference levels for magnetic fields of 1–100 kHz to 27 µT for general public exposure to magnetic fields of 3 kHz to 10 MHz and 100 µT for occupational exposure [13]. In addition, the World Health Organization has indicated that high-quality research is needed to assess the biological effects of exposure to IF magnetic fields because there is limited experimental evidence to support the new reference level [14].

In this study, we investigated the effects of exposure to an IF magnetic field of 23 kHz and 2 mT_rms_ (*i.e.*, ~74-times the reference level in the new ICNIRP guidelines) on neutrophils, cells that are critical components of the immune system. The human immune system eliminates exogenous materials such as invading foreign microbes in order to maintain homeostasis. Neutrophils are the vital gatekeepers of the host microbiome, playing an important role in defending against invading microbes. In this study, we investigated chemotaxis and phagocytosis in human neutrophils differentiated from HL-60 cells exposed to an IF magnetic field.

## 2. Materials and Methods

### 2.1. Cell Culture

**Figure 1:**
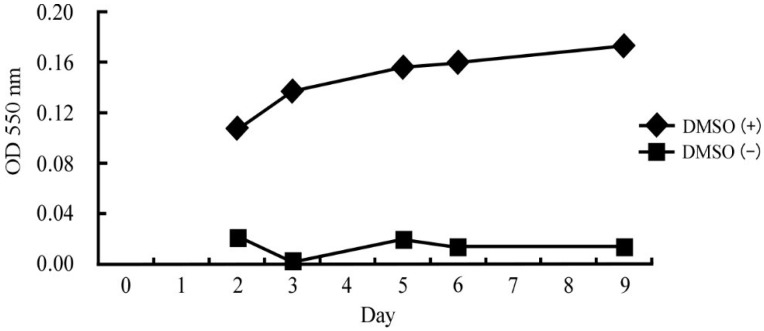
OD value at 550 nm, used to estimate the amount of superoxide in untreated and DMSO-treated HL-60 cells.

Human leukemia HL-60 cells (JCRB0085) were purchased from Japan Health Sciences Foundation (Tokyo, Japan) and were cultured in Roswell Park Memorial Institute (RPMI) 1640 medium supplemented with 10% inactivated fetal calf serum at 37 °C in an atmosphere of 95% air and 5% CO_2_. We previously optimized the conditions for differentiation of HL-60 cells [15]. Briefly, 1.25% dimethyl sulfoxide (DMSO) was added to 5 × 10^6^ cells in 38.1 mL of culture medium and the cells were incubated for 2–9 days. Figure 1 shows the change in superoxide (a differentiation marker of HL-60 cells) production over time after treatment with DMSO. The cells differentiated into neutrophil-like cells after 5 days of DMSO treatment.

### 2.2. Magnetic Field Generation System

After the cells had differentiated for 3 days, they were exposed to a 23-kHz IF magnetic field for 2, 3, or 4 h using a specially designed exposure apparatus. Our custom-built IF magnetic field generation system has been described elsewhere [16,17]. Briefly, this system consists of a custom-built magnetic generating coil, a power unit (KZ-MS32A; Panasonic, Osaka, Japan), a cooling unit (Carry Cool LPA3; Orion Machinery Co., Nagano, Japan), a temperature-monitoring system comprised of an optical-fiber thermometer (UM14; FISO Technology, Quebec, QC, Canada) with a data acquisition/switch unit (34970A; Agilent Technologies, Palo Alto, CA, USA), an incubator (modified BNR-110; Espec Corp., Osaka, Japan), and a water-circulating unit (modified Neo Cool Circulator CFA610; Yamato Scientific Co., Tokyo, Japan). The power unit was regulated to supply a current to the coil of 6.1 A_rms_ at 23 kHz to generate an IF magnetic field of 2 mT_rms_ from the coil. The magnetic field had a sinusoidal wave-form, with a vertical and uniaxial orientation. The magnetic flux density in the exposure space was measured using an ELT-400 tester (Narda STS, Pfullingen, Germany), with a 3-cm^2^ probe [16,17]. The temperature of the medium in the culture dishes was monitored throughout the experiment and maintained at 37.0 ± 0.5 °C. Sham exposure was performed using the same unit without IF magnetic field exposure.

### 2.3. Neutrophil Migration Capability

Neutrophil chemotaxis was assessed using an EZ-TAXIScan chemotaxis apparatus (ECI, Inc., Tokyo, Japan) [18]. Immediately after the exposure, the cells were collected and counted. A 1-μL volume of cells at a concentration of 2 × 10^6^/mL was applied to one of two compartments in the apparatus. Formyl-methionyl-leucyl-phenylalanine (fMLP) was added to the other compartment as a chemoattractant at a concentration of either 10^−7^ or 10^−8^ M. Time-lapse images were recorded every 30 s for 30 min, and cell migration was analyzed using a TAXIScan Analyzer2 (ECI, Inc., Tokyo, Japan). The migration velocity and directionality of cell movement were assessed for 20 cells per experiment, from which averages were calculated. The velocity of cell migration was expressed as μm/s, and the directionality of migration was expressed as the angle (radian) taken toward the chemoattractant from the start line (*i.e.*, π/2 indicates that the cells migrated straight toward the chemoattractant, and the value can be translated as 1.57).

Neutrophil migration capability was also assessed according to a second criterion, as calculated using the following formula:

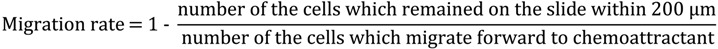
(1)

Values were calculated using Equation (1) 30 min after the addition of chemoattractant. This Equation (1) indicates the ratio of cell migration. We focused on the cells that migrated more than 30 μm forward toward the chemoattractant. The denominator describes the living cells to migrate to the chemoattractant. When the cells are active enough to migrate to the chemoattractant, they are able to reach the opposite side and cannot be seen within 200 µm. While the numerator describes the remained cells which cannot reach the chemoattractant though they are alive to move more than 30 μm. If the cells remain within 200 µm, the ratio of the right hand will larger. Then the Equation (1) indicates the migration rate of the cells.

### 2.4. Phagocytosis

Phagocytosis was evaluated using flow cytometry according to previously published methods [19,20,21]. Immediately after the exposure, the cells were collected and counted. A 100-μL volume of cells at a concentration of 1 × 10^4^/μL were incubated with 10 μL of FluoSheres fluorescent microspheres (1 × 10^10^ microspheres/mL; #F13081, Invitrogen, Carlsbad, CA, USA) for 40 min at 37 °C. Following ingestion of the microsphere particles by the neutro phils via phagocytosis, the cells were washed with PBS 5 times to remove any free particles and then resuspended with 1 mL of PBS and analyzed by flow cytometry (Nippon Becton Dickinson Company, Ltd., Tokyo, Japan).

### 2.5. Statistical Analysis

Data are expressed as the mean ± SD. Each experiment was performed at least three times, and the statistical significance of differences between sham and IF magnetic field exposure was assessed using a *t*-test. A *p*-value < 0.05 was considered to indicate statistical significance.

## 3. Results

### 3.1. Effect of IF Magnetic Field Exposure on Neutrophil Chemotaxis

**Figure 2:**
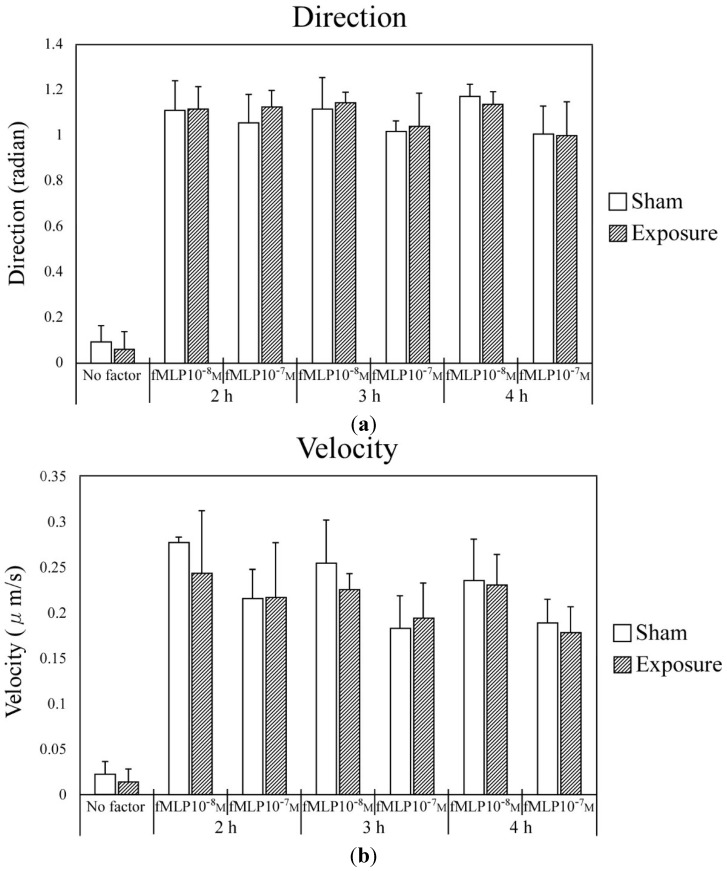
Chemotaxis of differentiated HL-60 cells after sham or IF magnetic field exposure for 2, 3, or 4 h. The directionality (**a**) and migration velocity (**b**) of differentiated HL-60 cell movement was assayed using 0, 10, or 100 nM fMLP as the chemoattractant.

The effect of exposure to IF magnetic fields on neutrophil chemotaxis was investigated using an EZ-TAXIScan chemotaxis assay system, which is widely used in research [22,23,24]. Figure 2 shows the effect of IF magnetic field exposure on neutrophil chemotaxis directionality and migration velocity. Exposure to a 23-kHz IF magnetic field had no effect on either the migration velocity or directionality of neutrophil chemotaxis. Addition of fMLP, which stimulates chemotaxis, significantly enhanced the migration velocity and directionality of chemotaxis compared with no addition of fMLP. However, the enhancement of migration velocity and directionality was not observed when fMLP addition was combined with exposure to the IF magnetic field. Longer duration of exposure to the IF magnetic field (e.g., 2, 3, or 4 h) also did not enhance chemotaxis.

**Figure 3:**
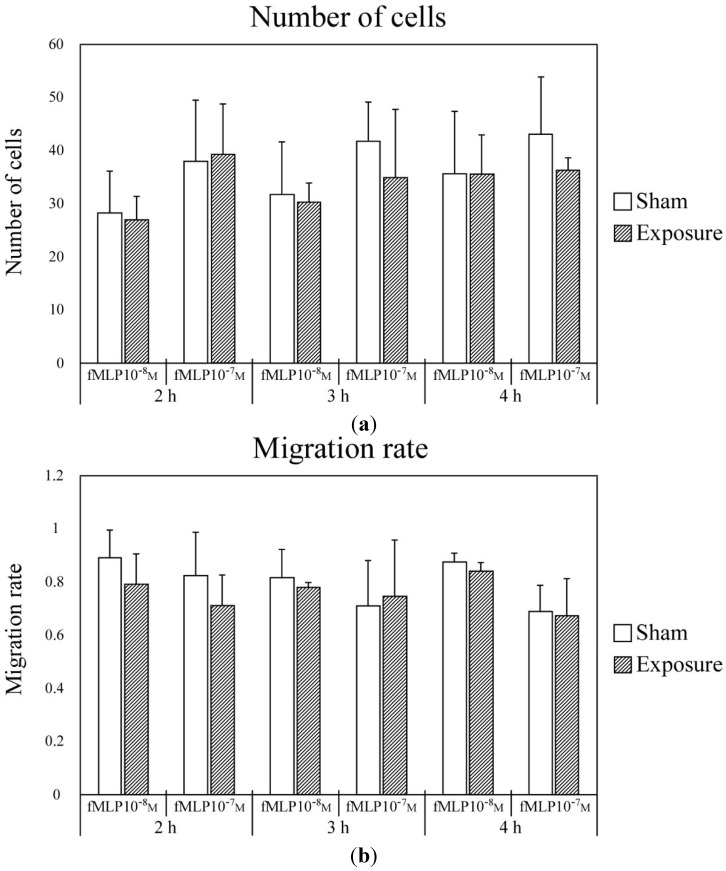
Chemotaxis of differentiated HL-60 cells was assayed using 10 or 100 nM fMLP as the chemoattractant after sham or IF magnetic field exposure for 2, 3, or 4 h. (**a**) Number of differentiated HL-60 cells migrating toward 10 or 100 nM fMLP as the chemoattractant. (**b**) Rate of migration of differentiated HL-60 cells toward 10 or 100 nM fMLP as the chemoattractant.

Figure 3a shows the number of neutrophils that migrated toward various concentrations of fMLP after sham or IF magnetic field exposure for varying periods of time. About 30 to 40 of these cells were evaluated for calculation of the migration rate in each experiment. Determination of the rate at which neutrophils migrated toward the various concentrations of fMLP after sham or 23-kHz IF magnetic field exposure for varying periods of time (Figure 3b) showed that exposure the IF magnetic field had no effect.

### 3.2. Effect of IF Magnetic Field Exposure on Phagocytosis

**Figure 4:**
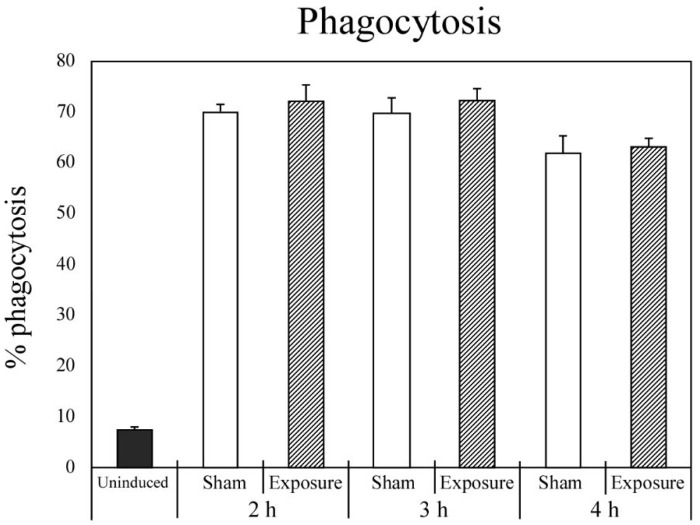
The percentage of microspheres phagocytosed by differentiated HL-60 cells after sham or IF magnetic field exposure for 2, 3, or 4 h.

Figure 4 shows the percentage of fluorescent microparticles phagocytosed by neutrophils exposed to a 23-kHz IF magnetic field for 2, 3 or 4 h, as determined by flow cytometry. The percentage of microparticles phagocytosed by cells exposed to the IF magnetic field was not significantly different from the percentage phagocytosed by cells that underwent sham exposure.

## 4. Discussion

In this study, we evaluated the effects of exposure to an IF magnetic field on chemotaxis and phagocytosis in human HL-60 cells using an EZ-TAXIScan chemotaxis apparatus and FluoSpheres fluorescent microspheres, respectively. We observed no differences between sham exposure and exposure to the IF magnetic field with respect to neutrophil migration and phagocytosis under all conditions investigated.

The results of previous research at the cellular level in our laboratory suggest that exposure to IF magnetic fields generated by IH cooktops is not genotoxic and has no effect on the expression of heat shock proteins [8,9,11,17]. In a 2007 study, we observed no genotoxic effects in bacteria or Chinese hamster ovary (CHO-K1) cells exposed to an IF magnetic field of 23 kHz at 532 ± 20 μT for 2 h [9]. Mutagenicity resulting from IF magnetic field exposure was examined in five different bacteria, and no significant difference in the frequency of revertant colony formation were observed between sham- and IF magnetic field-exposed bacteria. Compared with sham exposure, IF magnetic field exposure also had no effect on cell growth, micronucleus formation, comet assay results, and hypoxanthine guanine phosphoribosyl transferase (HPRT) gene mutations in CHO-K1 cells. We also reported that the expression of heat shock proteins in CHO-K1 cells is not affected by exposure to an IF magnetic field at 6.05 mT [11], an intensity higher than the 2 mT used in the present study. As these studies involved bacteria or hamster cells rather than human cells, we then evaluated the effects of IF magnetic field exposure on fetus-derived human SVGp12 astroglial cells [8]. We found that exposure to high- or low-intensity magnetic fields had no effect on gene expression in SVGp12 cells [17]. These data are in agreement with the findings of the present study.

In the present study, we did not detect any differences in neutrophil chemotaxis or phagocytosis following IF magnetic field exposure. Neutrophil chemotaxis was evaluated using fMLP as a chemoattractant. Obvious differences in migration were observed with and without chemoattractant; however, there was no difference between sham and IF magnetic field exposure. Although uninduced cells exhibited minimal phagocytic activity, the differentiated cells phagocytosed a significant percentage of the microspheres. However, no difference in phagocytic activity was observed between sham-exposed and IF magnetic field-exposed cells. These results confirmed that our experimental procedures were performed appropriately.

Few studies have evaluated the effects of exposure to IF magnetic fields on cells. However, some reports [25,26,27] have suggested that IF magnetic fields may have biological effects in animals. Blank *et al.* [28] reported that magnetic fields in the range 10 to 2500 Hz increase the activity of cytochrome oxidase, which may have biological effects at the cellular level. Although there are some negative data regarding the effects of exposure to IF magnetic fields [29,30], the results are inconsistent.

Although we demonstrated in this study that exposure to a 23-kHz IF magnetic field at 2 mT has no effect on chemotaxis and phagocytosis in differentiated HL-60 cells, further investigation is needed to elucidate the relationship between IF magnetic field exposure and human health. Our results suggest that exposure to a 23-kHz IF magnetic field at 2 mT (74-times the maximum value recommended by ICNIRP guidelines [12]) has no adverse effect on neutrophils, cells that play a critical role in the initial immune response to potential pathogens in humans.

## 5. Conclusions

Although the number of experiment was limited for the statistical analysis in this study, our results indicated that exposure to a 23-kHz IF magnetic field has no or very little effect on chemotaxis and phagocytic activity in neutrophil-like differentiated human HL-60 cells.
